# Intraocular pressure changes after phacoemulsification in pseudoexfoliation versus healthy eyes

**DOI:** 10.1186/s12886-021-01970-y

**Published:** 2021-05-06

**Authors:** Farshid Ramezani, Mohammad Nazarian, Leila Rezaei

**Affiliations:** Clinical Research Development Center, Imam Khomeini and Mohammad Kermanshahi and Farabi Hospitals, Kermanshah University of Medical Sciences, Kermanshah, Iran

**Keywords:** Pseudoexfoliation syndrome, Intraocular pressure, Phacoemulsification

## Abstract

**Background:**

Pseudoexfoliation (PXF) syndrome is the most common cause of secondary glaucoma worldwide. This systemic disorder causes further damage to the optic nerve and ultimately increases the need for surgical interventions. Therefore, intraocular pressure (IOP) control is very important in these patients. The aim of this study was to compare IOP changes after phacoemulsification in subjects with PXF syndrome compared to those without this syndrome.

**Methods:**

61 patients were enrolled in this prospective clinical study. Subjects were assigned into two groups based on presence or absence of PXF syndrome. IOP and anterior chamber angle parameters including: angle opening distance (AOD) and trabecular-iris surface area (TISA) measured one day preoperatively and 3 months postoperatively. Intraoperative metrics factors including: infusion fluid usage (IFU), cumulative dissipated energy (CDE) and aspiration time (AT) were obtained from the phacoemulsification machine at the end of each surgery. IOP changes, anterior chamber angle parameters and intraoperative metrics factors were compared between groups.

**Results:**

Mean IOP before surgery was significantly higher in the PXF group (14.70 mm Hg) compared to controls (12.87 mm Hg) (*P*-value < 0.01). Phacoemulsification decreased IOP in both, but to greater extent in the PXF group (*p*-value < 0.01). AOD and TISA also increased significantly following surgery in both groups. The results showed that postoperative IOP was negatively correlated with preoperative IOP in both groups (*p*-value < 0.01). Also, IOP after phacoemulsification was negatively correlated with IFU in the PXF group (*p*-value = 0.03).

**Conclusions:**

Patients with PXF syndrome exhibited a reduction in IOP and increase in anterior chamber angle parameters after phacoemulsification. We observed a greater IOP reduction in PXF subjects when it was compared to controls. Higher preoperative IOP and intraoperative IFU were associated with more IOP reduction in the PXF group.

## Background

Pseudoexfoliation (PXF) syndrome is a systemic disorder characterized by accumulation of gray flaky material on various parts of the eye such as the cornea, trabecular meshwork (TM), lens, pupillary margin, ciliary body, iris and blood vessels. This syndrome may affect other organs such as the heart and skin, but its main manifestation is in the eye [1].

PXF syndrome is the most common cause of both secondary and unilateral glaucoma. About 25 % of PXF subjects exhibit increased intraocular pressure (IOP) which may be associated with glaucomatous damages [2, 3]. Compared to primary open angle glaucoma (POAG), patients with PXF syndrome show more severe optic nerve and visual field damages, lower response to medical treatments and rapid glaucoma progression. Moreover, these patients undergo more frequent surgical interventions [4].

Predicting the precise level of IOP after phacoemulsification is critical to selection of an appropriate intervention approach such as: phacoemulsification, glaucoma surgery or a combined procedure.

Recent studies have reported significant IOP reduction after cataract surgery in patients with ocular hypertension (OHT), glaucoma and also in non-glaucomatous patients [5–13]. Damji et al. reported that using higher volumes of irrigation fluid during phacoemulsification was significantly associated with a greater IOP reduction in PXF subjects [14]. In a report by the American Academy of Ophthalmology, it was shown that phacoemulsification resulted in IOP reduction and reduce of medications in patients with PXF glaucoma, POAG and primary angle closure glaucoma (PACG) [15]. Moghimi et al. showed that non-glaucomatous patients with PXF syndrome experienced moderate IOP reduction after phacoemulsification which was correlated with preoperative IOP, infusion fluid usage (IFU) and intraoperative aspiration time (AT) [16].

Anterior segment optical coherence tomography (ASOCT) is a non-contact, rapid imaging of anterior segment anatomy. It measure parameters such as the anterior chamber depth and angle’s width that allows ophthalmologist to document changes in anterior segment structures [17].

Today’s phacoemulsification machines can record different intraoperative metric parameters, including IFU, AT and cumulative dissipated energy (CDE). CDE is the amount of the ultrasound energy applied within the eye during phacoemulsification.

The aim of this study was to compare IOP changes after phacoemulsification in subjects with and without PXF syndrome. Moreover, the effects of anterior segment parameters (measured by ASOCT) and intraoperative metric factors (obtained from the phacoemulsification machine) on IOP changes were evaluated.

## Methods

This prospective clinical study was performed on patients with cataract who were divided into two groups based on the presence or absence of PXF syndrome. Patients were referred to the Ophthalmology Clinic of Imam Khomeini Hospital in Kermanshah City from Jan. 2019 to Jan. 2020.

The Ethical Review Committee of Kermanshah University of Medical Sciences approved the study. Informed written consent was taken from all participants. It was performed in accordance with the tenets of the Declaration of Helsinki.

Inclusion criteria included those with obvious evidence of white or gray PXF deposits on anterior lens surface or pupillary margin (PXF group), visually significant cataract (best corrected visual acuity < 20/30), normal appearance of the optic nerve head (ONH) without cupping, rim notching or pallor and normal results of standard automated perimetry.

Patients with previous laser treatment or eye surgery, IOP > 30 mm Hg, history of elevated IOP and those unable to give consent or participate in follow-up sessions were excluded.

Ophthalmic examinations were performed for all patients one day prior to cataract surgery, including; slit lamp examination, IOP measurement (by Goldmann applanation tonometry), visual acuity testing, gonioscopy (according to the Schaffer grading system) using a Zeiss-style 4-mirror goniolens, anterior chamber depth (ACD) and central corneal thickness (CCT) measurement by Optical biometry (Haag-Streit Lenstar LS900) and Humphrey visual field testing (central 24 − 2 SITA standard program; Carl Zeiss).

Reliability indices of standard automated perimetry results were rates of fixation loss, false negatives < 25 % and false positives < 15 %.

Given the notion that ocular inflammation in the early phase after cataract surgery may lead to IOP fluctuation, we used IOP measured three months after surgery for analysis. Average value of IOP was obtained from 2 measurements during preoperative and postoperative examination. If there was a difference of more than 2 mm Hg, a third IOP measurement was performed and the average of 3 measurements was used in final analysis.

### Surgical technique

All patients underwent clear corneal incision phacoemulsification. The same phacoemulsification machine was used in all cases (Stellaris; Bausch & Lomb). All surgeries were performed by one experienced surgeon under topical anesthesia. Eyes were prepared for surgery by instilling tropicamide 0.5 % and phenylephrine 10 % for pupil dilation. The surgeon first created a paracentesis and injected viscoelastic into the anterior chamber. A temporal 3 mm wide by 2 mm long clear corneal incision was made. Surgery consisted of injecting viscoelastic material into the anterior chamber, continuous curvilinear capsulorrhexis, hydrodissection, in-the-bag phacoemulsification using the chop technique, cortex aspiration, additional injection of viscoelastic material, and insertion of a foldable acrylic IOL (Akreos Adapt AO; Bausch & Lomb) in the capsular bag. The viscoelastic material was then removed. The corneal incision was closed by hydration. After the surgery, intraoperative metrics (including CDE, IFU and AT) were obtained from phacoemulsification machine. Follow-up examinations were carried out at one day, one week, one month and 3 months postoperatively.

All ASOCT imaging was done by the same trained operator one day before and 3 months after surgery. Images were excluded if the scleral spurs could not be identified. The anatomic angle parameters including AOD and TISA were measured. AOD500 Involves measuring a distance between the corneal endothelium and the iris at 500 μm anterior to the scleral spur. TISA500 is a trapezoidal area bordered by the AOD500, a line drawn from the scleral spur perpendicular to the iris, the inner corneoscleral wall, and the anterior surface of the iris [18, 19]. In this study AOD500 and TISA500 were mentioned as AOD and TISA.

### Statistical analysis

IOP change after 3 months was the main variable in this study. The paired t-test was used to compare mean IOP before and after surgery. Normal and non-normal distributions were determined by Kolmogorov-Smirnov and Shapiro-Wilk tests. Non-parametric tests were used for data that were not normally distributed. We used the Spearman’s rho correlation coefficient to study the relation between IOP changes from baseline and preoperative characteristics. Statistical analysis was performed using SPSS for windows (Version 26). A *P*-value < 0.05 was considered statistically significant.

## Results

70 eyes of 70 patients were enrolled in the study and underwent cataract surgery. We followed the patients for 3 months. Among them, five patients were excluded, as they did not attend the follow up examinations. In addition, four patients were excluded from the study due to poor-quality ASOCT images. Therefore, 61 eyes of 61 patients were included in final analysis (30 PXF group and 31 controls). The mean age was 69.00 ± 7.60 and 59.16 ± 10.28 years in the PXF group and controls, respectively. Table 1 summarizes the clinical characteristics of the cases.

**Table 1:** Clinical characteristics of the study population and average recorded intraoperative metrics during the surgery

	PXF Group	Control Group	*P*-value (Mann-Whitney)
Number of patients	30	31	-
Number of eyes	30	31	-
Right eye/Left eye	16/14	14/17	-
Male/Female	14/16	18/13	-
Narrow angle/Open angle	12/18	9/22	-
Mean ages ± SD in years	69.00 ± 7.60	59.16 ± 10.28	< 0.01
Mean preoperative gonioscopy grade (by Shaffer classification) ± SD	2.50 ± 0.57	2.74 ± 0.77	0.08
Mean ACD ± SD in mm	3.19 ± 0.22	3.21 ± 0.40	0.89
Mean CCT ± SD in μm	544.23 ± 35.05	521.54 ± 35.39	0.03
Mean Pre-op IOP ± SD in mm Hg	14.70 ± 2.42	12.87 ± 2.66	< 0.01
Mean Post-op IOP ± SD in mm Hg	10.20 ± 1.34	10.51 ± 2.11	0.94
Mean Pre-op AOD ± SD in μm	363.80 ± 107.87	407.19 ± 162.28	0.08
Mean Pre-op TISA ± SD in μm²	0.13 ± 0.04	0.15 ± 0.05	0.06
Intraoperative metrics			
Mean CDE ± SD in percent-second	9.24 ± 4.64	9.50 ± 6.39	0.72
Mean IFU ± SD in mL	139.00 ± 40	134.10 ± 49.13	0.46
Mean AT ± SD in minute	2.67 ± 0.76	2.16 ± 0.74	0.03

*ACD* anterior chamber depth, *AOD* angle opening distance, *AT* aspiration time, *CCT* central corneal thickness, *CDE* cumulative dissipated energy, *IFU* infusion fluid usage, *IOP* intraocular pressure, *Post-op* postoperative, *Pre-op* preoperative, *PXF* pseudoexfoliation, *SD* standard deviation, *TISA* trabecular-iris space area

### Intraocular pressure before and after Phacoemulsification

Prior to cataract surgery the mean IOP were 14.70 and 12.87 mm Hg in PXF and control groups, respectively, that reduced to 10.20 and 10.51 mm Hg after Phacoemulsification (Table 2). The mean preoperative IOP was significantly higher in the PXF group (Table 1). Also, Phacoemulsification reduced mean IOP in both groups which this reduction was significantly greater in the PXF group (*p* < 0.01).

**Table 2:** Intraocular pressure changes in the PXF and control groups

	1 day Pre-op IOP (mm Hg)	3 months Post-op IOP (mm Hg)	*P*-value (Paired t-test)
**PXF Group**	14.70	10.20	< 0.01
**Control Group**	12.87	10.51	< 0.01

*IOP *intraocular pressure, *Post-op *postoperative, *Pre-op *preoperative, *PXF *pseudoexfoliation,

### Intraocular pressure differences before and after Phacoemulsification

Herein, IOP-Diff refers to the difference between pre and postoperative IOP. The mean IOP-Diffs were − 4.50 and − 2.35 mmHg in PXF and control groups, respectively. The results showed that the absolute value of IOP-Diff was significantly higher in the PXF group when it was compared to the controls (Table 3).

**Table 3:** comparison of IOP-Diff and Relative IOP changes between PXF and Control Groups

	PXF Group	Control Group	*P*-value (Mann-Whitney)
**IOP-Diff (mm Hg)**	-4.50	-2.35	< 0.01
**Relative IOP changes**	-0.29	-0.16	< 0.01

*IOP *intraocular pressure, *IOP-Diff *intraocular pressure difference, *PXF *pseudoexfoliation,

### Relative changes in intraocular pressure before and after Phacoemulsification

The relative changes in IOP were obtained by dividing IOP-Diff by preoperative IOP. The mean relative changes in IOP were − 0.29 and − 0.16 in PXF and control groups, respectively. The absolute value of this change was significantly higher in the PXF group when it was compared to the controls (Table 3).

### The effect of preoperative intraocular pressure on intraocular pressure changes after phacoemulsification

We assessed the effect of preoperative IOP on the absolute value of IOP changes after surgery in all subjects. The analysis showed a statistically significant correlation between preoperative IOP and absolute value of IOP changes in PXF and control groups. The correlation coefficient values were + 0.84 and + 0.71, respectively (*p* < 0.01). Meaning that there was a positive association between preoperative IOP and IOP changes (Table 4). Similar results were obtained when assessing the effect of preoperative IOP on absolute value of relative IOP changes. The correlation coefficient values were + 0.70 and + 0.56 in PXF and control groups, respectively (*p* < 0.01).

**Table 4:** The effect of different variables before and during surgery on IOP changes in study groups

Variables	PXF Group		Control Group	
	**Correlation** **coefficient**	*P*-value (Spearman’s rho)	**Correlation** **coefficient**	*P*-value (Spearman’s rho)
**1 day Pre-op ACD**	-0.05	0.76	0.00	0.99
**1 day Pre-op TISA**	-0.05	0.72	-0.09	0.60
**1 day Pre-op AOD**	0.04	0.79	-0.11	0.52
**1 day Pre-op Angle**	0.11	0.53	-0.17	0.35
**1 day Pre-op IOP**	**0.84**	**< 0.01**	**0.71**	**< 0.01**
**AOD changes after 3 months**	-0.33	0.07	-0.15	0.39
**TISA changes after 3 months**	0.09	0.61	-0.17	0.34
**CDE**	-0.12	0.52	0.03	0.85
**AT**	0.07	0.72	0.08	0.66
**IFU**	**0.39**	**0.03**	**-0.52**	**< 0.01**

*ACD *anterior chamber depth, *AOD *angle opening distance, *AT *aspiration time, *CDE *cumulative dissipated energy, *IFU *infusion fluid usage, *IOP *intraocular pressure, *Pre-op *preoperative, *PXF *pseudoexfoliation, *TISA *trabecular-iris space area

### The effect of Intraoperative metrics factors on intraocular pressure changes after phacoemulsification

**Fig. 1:**
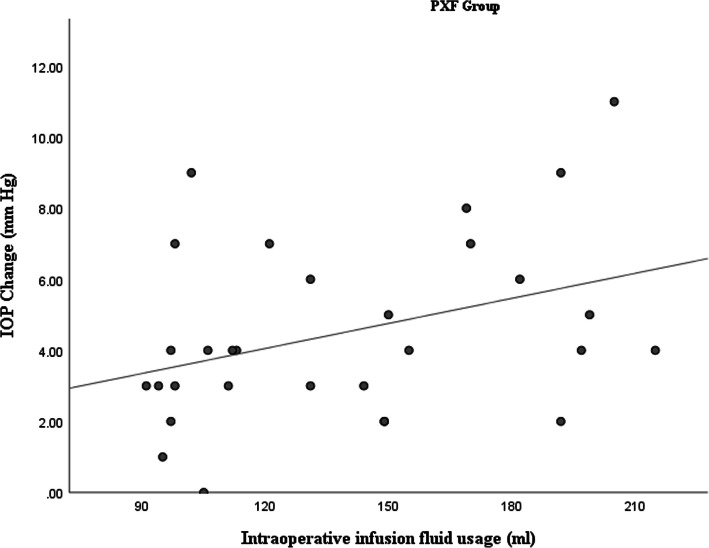
A scatter plot demonstrating positive correlation between infusion fluid usage and intraocular pressure changes after phacoemulsification in the pseudoexfoliation group

**Fig. 2:**
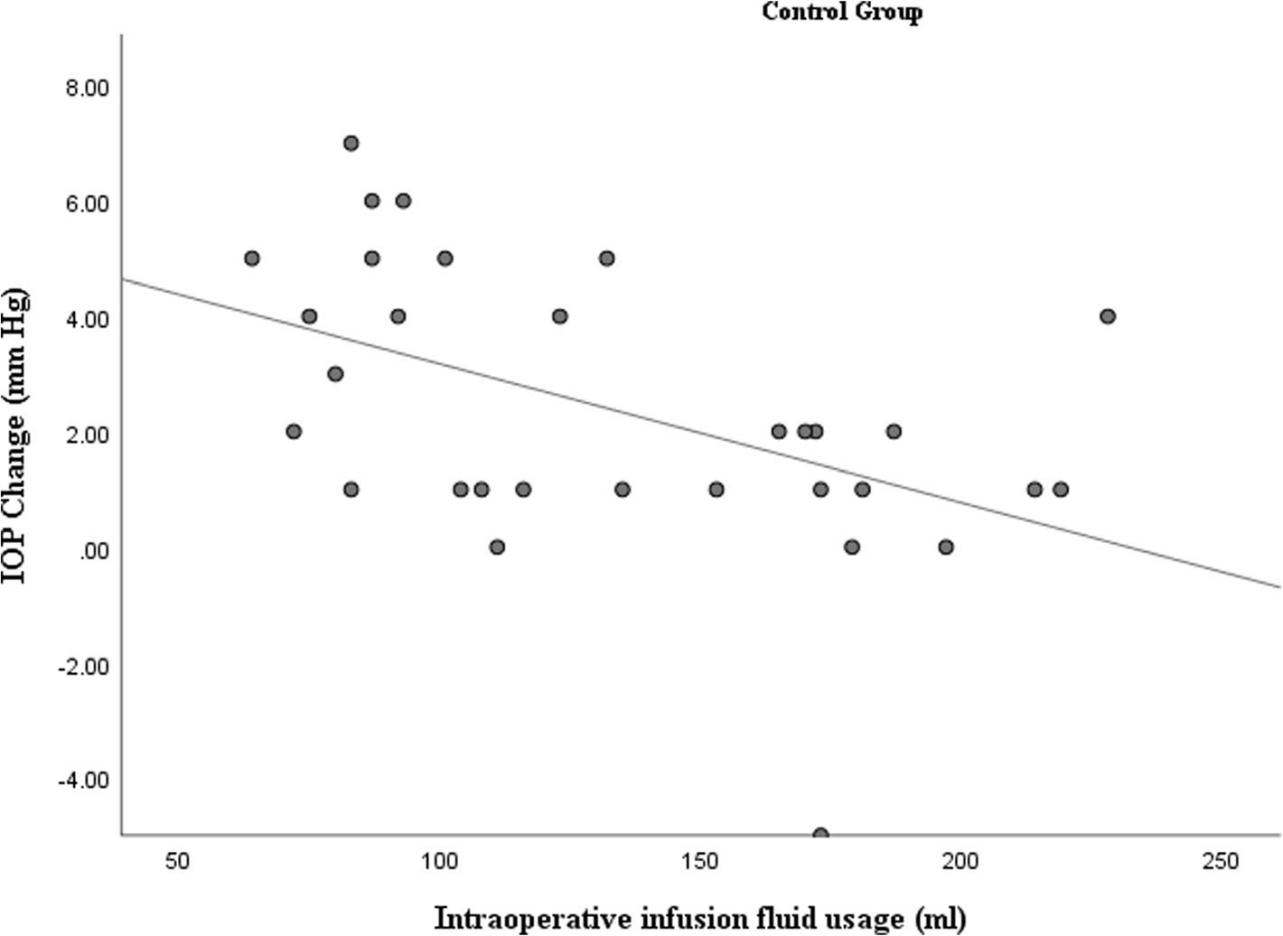
A scatter plot demonstrating negative correlation between infusion fluid usage and intraocular pressure changes after phacoemulsification in the control group

Intraoperative metrics factors including: IFU, CDE and AT were obtained from the phacoemulsification machine at the end of each surgery. We observed a statistically positive correlation between IFU and absolute value of IOP changes in the PXF group. The correlation coefficient value was + 0.39 (*p* = 0.03), pointing towards a positive association between IFU and IOP changes (Fig. 1). However, in the control group the correlation coefficient value between IFU and absolute value of IOP changes was − 0.52 (*p* < 0.01), indicating a negative association between IFU and IOP changes (Fig. 2).

Nearly similar results were obtained when assessing the effect of IFU on absolute values of relative IOP changes. The correlation coefficient values were + 0.33 (*p* = 0.07) and − 0.60 (*p* < 0.01) in PXF and control groups, respectively.

There was no significant correlation between AT or CDE and absolute value of IOP changes or relative IOP changes in either group (Table 4).

### Anterior chamber parameters changes before and after Phacoemulsification

The mean preoperative AOD were 363.80 and 407.19 μm in PXF and control groups, respectively, that increased to 591.30 and 590.26 μm after phacoemulsification. The results showed that the postoperative AOD was significantly increased in both Groups (*p* < 0.01). This effect was greater in the PXF group.

The mean preoperative TISA were 0.133 and 0.149 μm² in PXF and control groups, respectively, that increased to 0.198 and 0.197 μm² after phacoemulsification. The results showed that the postoperative TISA was significantly increased in both Groups (*p* < 0.01). This effect was greater in the PXF group.

### Other variables and intraocular pressure changes after Phacoemulsification

Anterior chamber angle parameters including: AOD and TISA were measured one day before surgery and three months after surgery. AOD and TISA changes were defined as postoperative AOD or TISA minus preoperative AOD or TISA. There was no significant correlation between anterior chamber angle parameters changes and IOP changes or relative IOP changes in either group (Table 4).

Moreover, we did not observe a significant correlation between preoperative ACD, anterior chamber angle (in terms of degree) and anterior chamber angle parameters (AOD and TISA) with IOP changes or relative IOP changes in either group (Table 4).

## Discussion

Ample evidence suggests that PXF syndrome is the most common detectable cause of glaucoma worldwide [20]. In our study, we observed higher Preoperative IOP in the PXF group compared to control group. Phacoemulsification reduced IOP in both groups which this effect was significantly greater in the PXF group.

To better compare the IOP changes in those two groups, we defined two variables; IOP-Diff: the IOP difference before and after phacoemulsification (IOP-Diff = Postoperative IOP – Preoperative IOP) and IOP-Diff Ratio: the relative change in IOP before and after surgery (IOP-Diff Ratio = IOP-Diff dividing by preoperative IOP). We described these two new variables to Better comparison of IOP changes in the two groups and reduce the possible confounding effect of preoperative IOP level on the study results. Absolute value of IOP-Diff and IOP-Diff Ratio was significantly higher in the PXF group when it was compared to the control group; Suggesting that individuals with PXF syndrome could experience a greater IOP reduction after phacoemulsification. Previous studies have examined IOP changes after phacoemulsification as ΔIOP [10, 14]. Damji et al. in a prospective study demonstrated that ΔIOP was significantly greater in eyes with PXF syndrome versus controls [14]. However, relative IOP changes have not been compared in those studies.

This study showed that higher preoperative IOP was associated with greater IOP changes and relative IOP changes after phacoemulsification. This means that Patients with higher preoperative IOP experience a greater reduction in postoperative IOP. Therefore, higher preoperative IOP could be a predictive factor for further reduction of postoperative IOP. Consistent with this result, several studies have showed preoperative IOP as a significant predictive factor for postoperative IOP reduction [16, 21–23].

The exact mechanism underlying the IOP reduction after cataract surgery is still unclear. Increasing aqueous drainage through the TM due to deepening of the anterior chamber has been suggested as a potential mechanism [24]. Alaghband et al. measured tonographic outflow facility (TOF) of the eye by electronic Schiotz tonography and showed that phacoemulsification increases TOF [25]. In patients with PXF syndrome, accumulation of exfoliation materials and pigments in the TM may lead to the TM obstruction and IOP elevation [1]. Therefore, surgical removal of these materials during phacoemulsification could lead to a greater IOP reduction in these patients. Moreover, phacoemulsification removes iridolenticular contact and thus reduces the release of pigment from the iris and exfoliation material from the lens and iris [14, 16].

Our study showed that with increasing infusion fluid during phacoemulsification, patients with PXF syndrome experience a larger IOP reduction after the surgery. However, in patients without PXF syndrome, an increase in infusion fluid during phacoemulsification was associated with a lower reduction in postoperative IOP. One may speculate that in patients with PXF syndrome, more infusion fluid during phacoemulsification is associated with more aspiration of pigments and exfoliation materials. This could lead to a further reduction in IOP after surgery. On the other hand, in patients without PXF syndrome, higher infusion fluid during phacoemulsification, which may indicate a longer operation time, could be associated with a higher risk of postoperative inflammation and a smaller reduction in IOP after the surgery.

Few investigations have studied the correlation between infusion fluid during phacoemulsification and postoperative IOP. Damji et al. demonstrated that the IOP lowering effect in the PXF group was related to irrigation volume at the time of surgery, but he did not show any correlation in the non-PXF group [14]. Moghimi et al. described that greater IFU during surgery led to greater IOP drop after phacoemulsification in eyes with PXF syndrome [16].

Anterior chamber angle parameters in patients with and without PXF syndrome increased significantly 3 months post operatively. This effect was greater in patients with PXF syndrome. These changes could be related to the removal of the crystalline lens effect after phacoemulsification. However, this effect was not correlated with IOP changes in both groups. In the present study, most patients were open angle before phacoemulsification and perhaps that is why there was no significant correlation between preoperative anterior chamber angle parameters and IOP changes after phacoemulsification. These results suggest that microscopic TM changes may be involved in the determination of IOP level other than macroscopic angle parameters in these eyes [16].

This study had some limitations that must be considered. The main limitations were the Small sample size, and a relatively short follow-up period. A longer follow-up would have allowed a better evaluation of the long-term effect of cataract surgery on postoperative IOP changes.

## Conclusions

Herein, we studied IOP changes after phacoemulsification in cataract patients with and without PXF syndrome. Our data demonstrated that reduction in IOP was significantly greater in patients with PXF syndrome. Moreover, higher preoperative IOP was associated with a greater IOP reduction after surgery in both groups after 3 months. In addition, higher intraoperative IFU was associated with more IOP reduction in the PXF group and less IOP reduction in the control group. Consequently, in cataract patients with PXF syndrome a rather lower threshold for performing phacoemulsification with a rather more IFU during surgery, could be considered. Of course, additional studies are necessary to confirm this.

## Data Availability

The datasets generated and analyzed during the current study are not publicly available due to their containing information that could compromise the privacy of research participants but are available from the corresponding author on reasonable request.

## Abbreviations

ACD: Anterior chamber depth
AOD: Angle opening distance
ASOCT: Anterior segment optical coherence tomography
AT: Aspiration time
CCT: Central corneal thickness
CDE: Cumulative dissipated energy
IFU: Infusion fluid usage
IOP: Intraocular pressure
OHT: Ocular hypertension
ONH: Optic nerve head
PACG: Primary angle closure glaucoma
POAG: Primary open angle glaucoma
PXF: Pseudoexfoliation
TISA: Trabecular-iris surface area
TM: Trabecular meshwork
